# Numerical Modelling of CFS Three-Story Strap-Braced Building under Shaking-Table Excitations

**DOI:** 10.3390/ma14010118

**Published:** 2020-12-29

**Authors:** Alessia Campiche

**Affiliations:** Department of Structures for Engineering and Architecture, University of Naples “Federico II”, Via Forno Vecchio 36, 80134 Naples, Italy; alessia.campiche@unina.it; Tel.: +39-081-2538982

**Keywords:** CFS structures, seismic design, strap-braced buildings, shake-table tests, numerical modelling, dynamic behavior

## Abstract

In recent research activities, shake-table tests were revealed to be useful to investigate the seismic behavior of cold-formed steel (CFS) buildings. However, testing full-scale buildings or reduced-scale prototypes is not always possible; indeed, predicting tools and numerical models could help designers to evaluate earthquake response. For this reason, numerical modelling of two strap-braced prototype buildings, recently tested on shake-table at University of Naples Federico II in cooperation with Lamieredil S.p.A. company, was developed. The models were validated trough the comparison between experimental and numerical results, in term of dynamic properties (fundamental period of vibration and modal shapes), peak roof drift ratios and peak inter-story drift ratios. Although dynamic properties of mock-ups were captured with accuracy by the developed models, the comparison highlighted the need to consider accumulation of damage and rocking phenomenon in the modelling to capture with good accuracy the seismic behavior of CFS strap-braced building, subjected to high intensity records.

## 1. Introduction

Seismic behavior of structures has been a key topic in the last decades and still under investigation. The University of Naples “Federico II” carried out research devoted to the evaluation of dynamic properties and seismic response of both traditional steel structures [1,2,3,4,5,6,7,8,9,10,11,12,13,14,15,16,17,18,19,20,21] and lightweight steel systems [22,23,24,25,26]. In this framework, an important research project funded by Lamieredil S.p.A. company, recently finished, which the main goal was the investigation of seismic behavior of lightweight steel (LWS) constructions and in particular cold-formed steel (CFS) strap-braced wall buildings. The structural part of these systems consists of CFS members’ frame braced by thin steel straps in X configuration, which the main task is the resistance to lateral loads. Although high performances and advantages of LWS constructions are now worldwide recognized, high-fidelity modelling tools easy-to-use for engineers have not been amply studied and widespread. Commonly, in the state of the art only the main lateral force resisting system (LFRS), i.e., strap-braced wall, is considered and two modelling approaches are employed: (i) A simplified approach in which the nonlinear behavior of steel straps is represented by a spring or a truss element [27,28,29,30]; (ii) detailed FE model using shell or frame elements [31,32,33,34] concerning different geometrical and material details. In the literature, few cases of CFS strap-braced wall buildings have been modelled [35,36,37], but there is no case in which experimentally tested buildings have been modelled and numerical results have been compared with experimental results.

To overcome this lack, starting from the experimental results, a specific task of the above-mentioned project was devoted to the development of 3D numerical models with OpenSees [38] software. In particular, monotonic and cyclic tests on full scale strap-braced walls and shake table tests on two reduced-scale (1:3) three-storiy CFS mock-ups, carried out at the lab of the Department for Structures and Engineering, were considered and used for the calibration and the validation of modelling choices. Firstly, the calibration of numerical models of CFS strap-braced stud walls was done on the base of the available results of quasi-static cyclic tests on single walls [39], then numerical models of whole mock-ups were developed in order to simulate the non-linear dynamic response.

After a brief introduction of mock-up geometries and shake-table tests, the paper provides the description of numerical modelling of the two tested mock-ups and the validation through the comparison with experimental results.

## 2. Mock-Up Description

**Figure 1:**
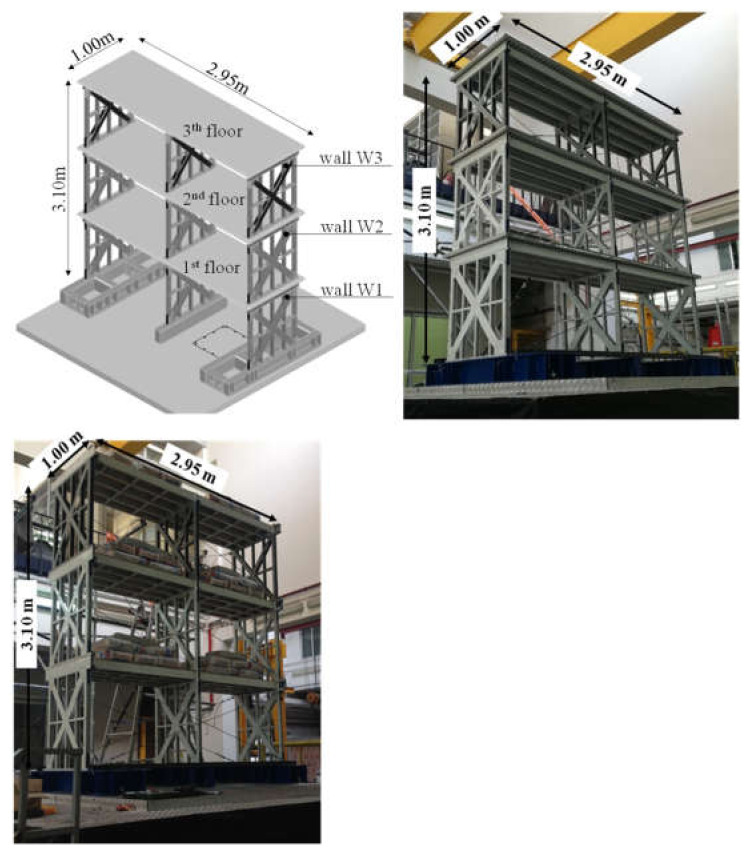
Reduced scale tested mock-ups [42].

The two tested mock-ups were reduced scale three-story and two bay CFS buildings (Figure 1) with a rectangular plan of 0.95 m × 2.95 m and a total height of about 3.10 m. The mock-ups were representative of two residential buildings designed and selected as case studies and they were scaled considering a scaling factor equal to 1:3. To scale the mock-ups, three independent scaling factors were fixed, length (S_L_ = 1/3), stress (Sσ = 1) and acceleration (S_A_ = 1), while scaling factors of other parameters (dependent parameters) are defined according to the similitude laws [40]. More details about case study design and scaling procedure are available in Fiorino et al. [41].

Both mock-ups were designed for gravity and seismic loads as “all-steel” solutions, neglecting the contribution of panels to the resistance, and they were mainly composed of strap-braced stud walls, as lateral force resisting systems (LFRSs), and floors. The only difference between the two mock-ups consisted of floor typology: (i) The first building, named Type 1 mock-up, had composite steel-concrete floors; (ii) the second building, named Type 2 mock-up, had wood-based panel floors. Strap-braced stud walls, having dimension 0.80 m × 0.90 m (length × height), were placed only in the short direction and were built with C studs, U tracks, and X diagonal flat straps with different dimensions for each story. For all the components as screws were used 2.2 mm × 9.5 mm self-piercing screws. All the elements used in the mock-ups are summarized in Table 1.

## 3. Shake-Table Tests

Dynamic and seismic response of the mock-ups were evaluated through a series of shake-table tests carried out at the lab of the Department of Structures for Engineering and Architecture of University of Naples “Federico II”.

White noise signals were used for the evaluation of dynamic properties (random test) of mock-ups in shorter direction, i.e., fundamental period of vibration and damping ratio. To analyze experimental data obtained, experimental modal analysis technique was used, thanks to ARTeMIS Modal software [43].

The natural ground motion record east–west acceleration component recorded by the Norcia (NRC) Station with a PGA of 4.76 m/s^2^ (0.49 g) opportunely scaled was used to evaluate the seismic response (earthquake test). Different earthquake record scaling factors were used to reproduce various ground motion hazard levels.

To track the spreading of damage through the variation in dynamic properties, earthquake tests were always preceded and followed by random tests. Table 2 shows the scaling factors used for earthquake records for both mock-ups.

Further details about shake-table tests are available in Fiorino et al. [41].

## 4. Numerical Modelling

To simulate the response of shake-table tests and provide a useful tool for next applications, three-dimensional finite element models of mock-ups were developed, using OpenSees software [38].

First of all, the LFRS modelling plays a key role to capture with accuracy the behavior of whole buildings. In the specific case, following the same approach adopted in [29], a pair of diagonal truss elements were used to model the LFRSs of the mock-ups, i.e., strap-braced stud walls, in which Pinching4 material, calibrated on the basis of cyclic test data [39], was used to simulate their hysteretic response. In particular, test data were used to calibrate the backbone envelope curves and the 23 parameters governing the cyclic behavior in Pinching4 material rule.

**Figure 2:**
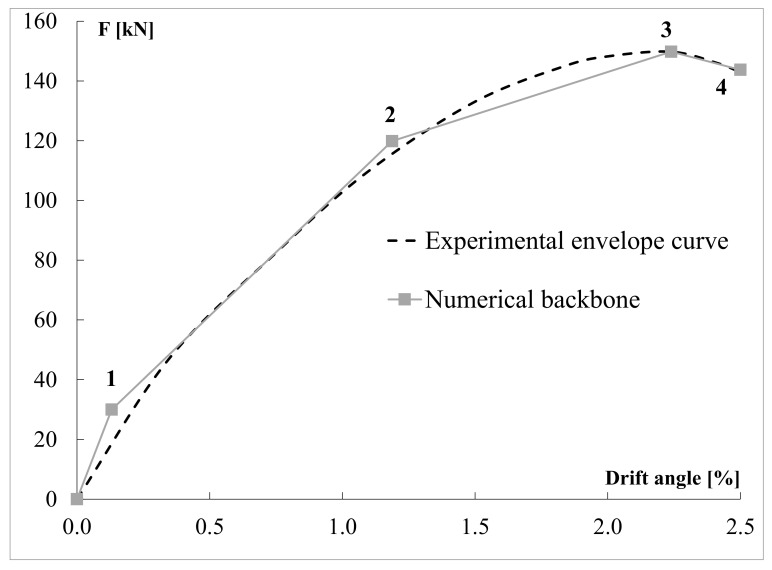
Comparison between experimental and numerical envelope curves for the wall located at third floor.

The first point (1) of the backbone envelope curves was located at 50% of the experimental yielding strength; the second point (2) corresponded to the elastic limit observed in experiments; the third point (3) represented the experimental peak strength; the fourth point (4) of numerical backbone curve corresponded to the maximum experimental displacement and the corresponding force, selected with an energy balance. In this way, the area under the numerical backbone curve between points (3) and (4) was equal to the corresponding area of the experimental envelope curve. The comparison between experimental and numerical envelope curves for the wall located at third floor is shown in Figure 2.

**Figure 3:**
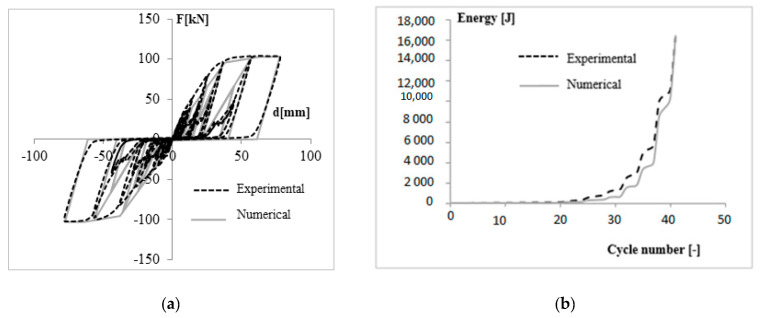
Modelling options and parameters used [42]. (**a**) Force-Displacement response of wall at third floor; (**b**) Cumulative energy dissipation of wall at third floor.

To define Pinching4 material many parameters need to control cyclic behavior. The parameter rDisp, governing ratio between the displacement of the reloading point and maximum positive displacement of preceding cycles, was set equal to 0.01, 0.05 and 0.1 for walls at first, second and third story, respectively. The parameter rForce was set equal to 0.01 for all the walls. Rest of the parameters required were set equal to 0. Wall models were validated by comparing the experimental [39] and numerical responses in term of force-displacement response (Figure 3a) and cumulative energy (Figure 3b). Since cyclic tests were carried out for all the wall configurations, each wall model was compared with the corresponding test. The comparison is shown in Figure 2 for the wall at third story.

**Figure 4:**
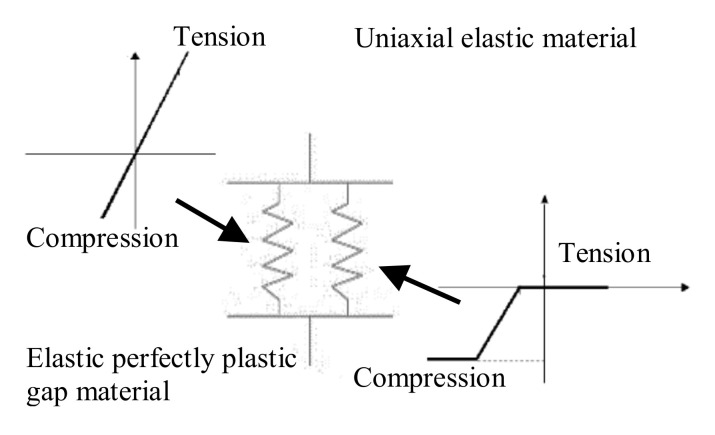
A two-node parallel spring element used for hold-downs [42].

Since the hold-down offers different stiffness in tension and compression, the approach suggested by Leng et al. [44] was employed; indeed, hold-downs were modelled with two parallel Zerolength elements (Figure 4), in which the first element used uniaxial elastic material representing the stiffness of hold-down connection only, while the second element used elastic-perfectly plastic gap material with a zero-gap, representing the stiffness in compression offered by foundation or floor. Uniaxial elastic material in case of the Type 1 mock-up had a stiffness of 20, 10, and 7.5 kN/mm for the walls at first, second and third story, respectively, whereas in case of Type 2 mock-up it had a stiffness of 20, 20, and 15 kN/mm for the walls at first, second and third story, respectively. Elastic-perfectly plastic gap material had stiffness of 20 kN/mm for foundation, whereas for floors a very low value was used. The yield strength used for Elastic-perfectly plastic gap material was also calibrated experimentally [39].

Infinite rigid truss elements were used to model the tracks, whereas truss elements with the uniaxialMaterial Elastic having the same properties of the profiles used in the mock-ups was employed for the chord studs. Buckling mechanisms and tensile rupture were explicitly considered in modelling.

According to the prescriptions provided in ASCE 7 [45], during the tests, floors behaved rigidly in their plane; indeed, the diaphragm model employed infinite rigid vertical and horizontal beam column elements. Moreover, since the motion of shake table was unidirectional, the motion in the other degree of freedom was restrained in the model.

**Figure 5:**
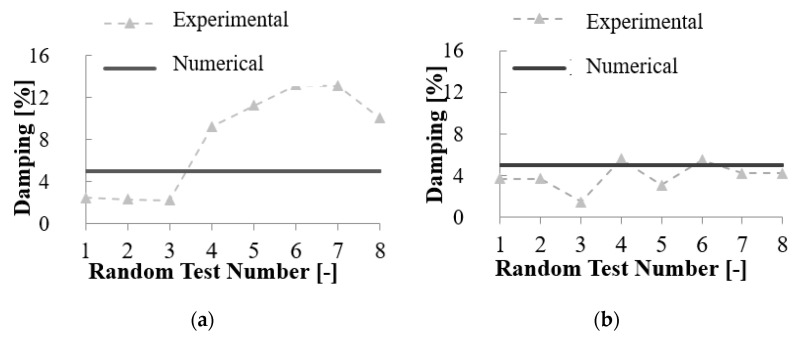
Experimental damping range for (**a**) Type 1 and (**b**) Type 2 mock-ups and Rayleigh damping value selected for the model.

**Figure 6:**
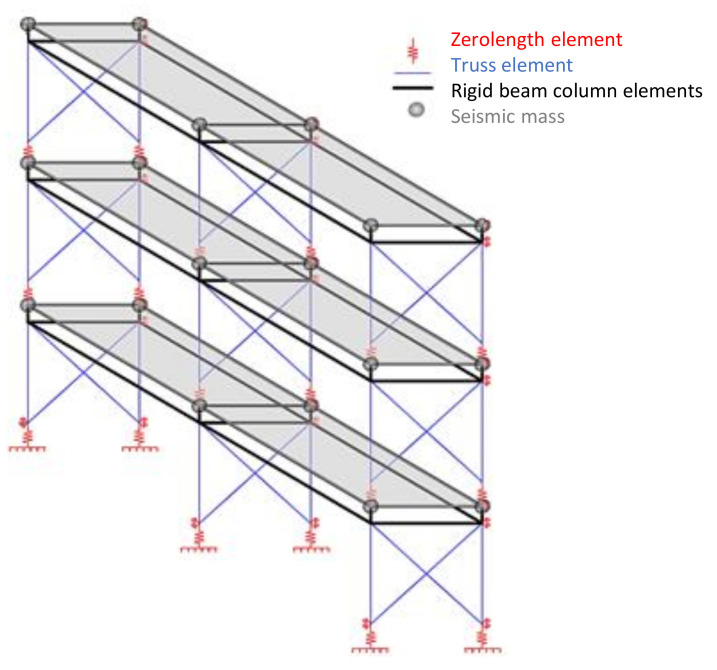
Schematic drawing of models developed.

Seismic mass of the buildings was concentrated at the top end nodes of walls at each floor. Damping was simulated by utilizing the Rayleigh damping. With regard to damping ratio, the experimental tests showed that it ranged from 2.4% (before Earthquake tests) to 13% for Type 1 mock-up, with an increasing of 5.4 times, and from 3.7% to 5.6% for the Type 2 mock-up, with an increasing of 1.5 times (Figure 5). For each model, a sensitivity analysis was carried out to select the best match for damping, which was selected equal to 5%. Figure 5 shows the experimental damping ratios measured during the random tests and the value of 5% chosen for the models; it is clear that 5% value matches the average experimental value obtained. The models were subjected to modal analysis and time history analysis with the inputs measured at the base of shake-table during the tests. Figure 6 shows a schematic drawing of the models developed.

## 5. Experimental vs. Numerical Results

In order to validate the models, a comparison among experimental and numerical results was conducted. In particular, dynamic identification results were compared in term of fundamental period of vibration and modal shapes, whereas the earthquake test results were compared in term of peak roof drift ratio (PRDR) and peak inter-story drift ratio (PIDR).

PRDR was defined as the ratio between the maximum top displacement and the height of the structure, whereas PIDR was defined as the ratio between the relative translational displacement difference between two consecutive floors and the story height.

**Figure 7:**
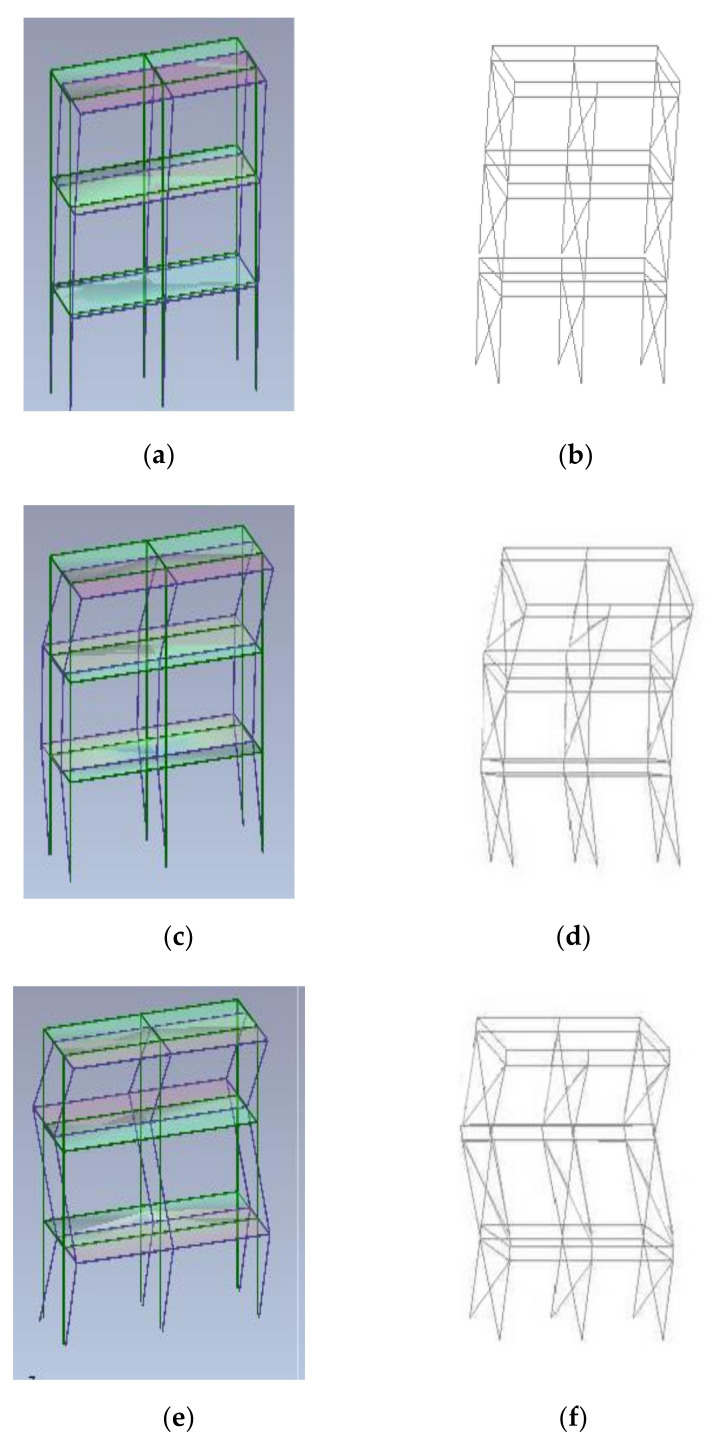
Comparison of experimental (Artemis) and numerical (OpenSees) modal shapes [42]. (**a**) First experimental modal shape; (**b**) First numerical modal shape; (**c**) Second experimental modal shape; (**d**) Second numerical modal shape; (**e**) Third experimental modal shape; (**f**) Third numerical modal shape.

The interpretation of experimental dynamic identification was performed through experimental modal analysis with ARTeMIS Modal software [43], whereas numerical dynamic identification consisted of modal analysis of models. The experimental results showed that the first period of vibration before Earthquake tests was 0.43 s and 0.50 s for the Type 1 and Type 2 mock-ups, respectively, whereas the numerical fundamental periods were 0.40 s and 0.50 s for Type 1 and Type 2 mock-ups, respectively (experimental-to-numerical ratio of 1.08 and 1.00 for Type 1 and Type 2 mock-ups, respectively). The periods of vibration corresponding to the second and third mode were also evaluated and compared. Comparison between experimental and numerical values are summarized in Table 3. The vibration modes obtained are shown in Figure 7. As a result, numerical models give a good prediction of dynamic properties of mock-ups for the first period of vibration, whereas they underestimate the second and third periods of vibration (experimental-to-numerical ratio in the range 1.25 to 1.43). This result could be mainly attributed to the presence of rocking phenomenon, which increases the actual deformability of the structures and the second and third modes of vibration are more sensitive than the first, since they have more inversions (Figure 7). Although rocking was experimentally observed in the shake-table tests, it was not well measured because ad-hoc instrumentation was not installed, since it has never happened before in previous experimental tests on strap-braced walls [23,29,39] or whole building [35].

**Figure 8:**
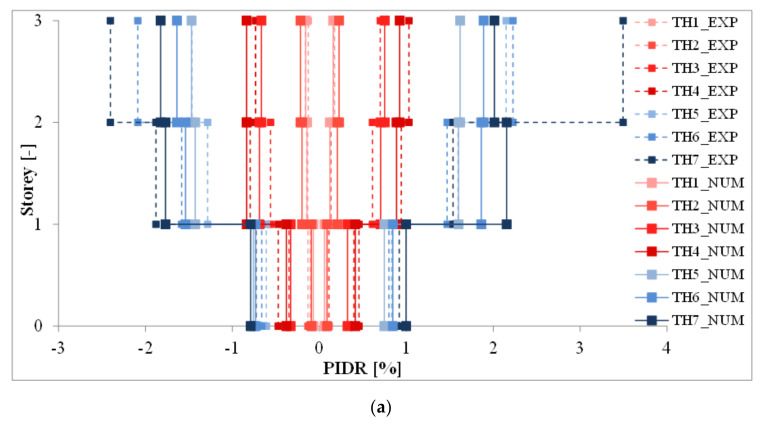

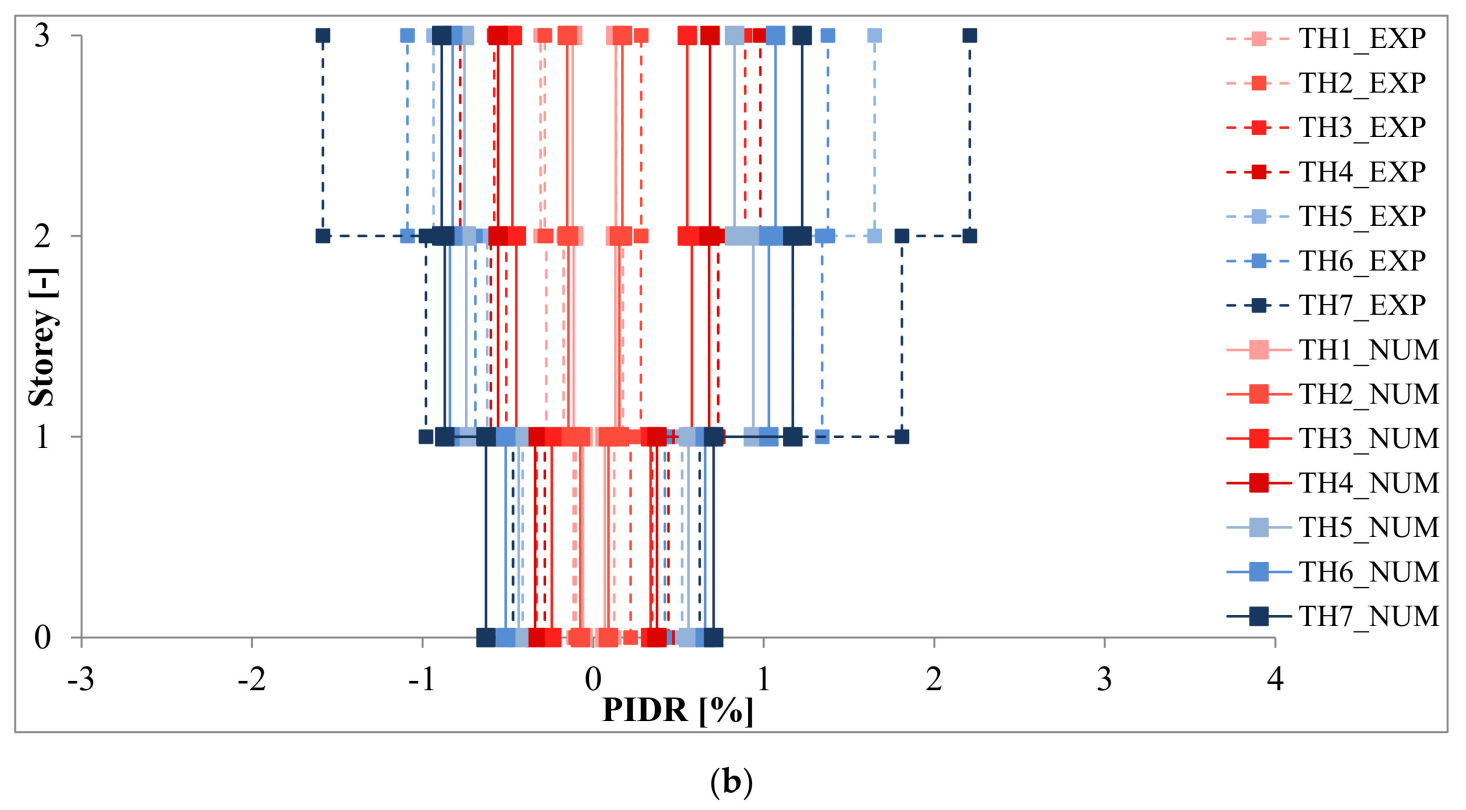
Comparison between experimental and numerical peak inter-story drift ratio (PIDR) [42]. (**a**) Type 1, (**b**) Type 2.

Non-linear time history analyses were performed to evaluate the numerical seismic response of mock-ups, that was compared with the experimental results to proof the goodness of models. As happened in the shake-table tests, the earthquakes were applied in sequence to the numerical models with increasing scale factors. In this way experimental and numerical results are more comparable. Numerical models also included the signal distortion, differences between signal really reproduced and signal imposed to shake-table. As concern the seismic response, PIDRs were reached for the input with maximum intensity (scaling factor of 150%) at the 3rd story: 3.49% for Type 1 mock-up and 2.21% for type 2 mock-up, whereas the PIDRs were 1.78% at 2nd level for Type 1 mock-up and 1.33% for Type 2 mock-up at 3rd level. A comparison between experimental and numerical results in terms of PIDR is given in Table 4. Figure 8 shows minimum and maximum inter-story drift ratios (IDRs) along the height of both mock-ups. The experimental to numerical ratio is in the range 0.80 to 2.08 for Type 1 mock-up and 0.65 to 2.06 for Type 2 mock-up. Models can capture PIDR for low inputs (until scaling factor of 100%), whereas they do not show good predictions for higher inputs (scaling factors of 120% and 150%). The reason behind the inaccurate response at high intensity earthquakes could be due to the high rocking phenomenon, which represents an adding source of deformability of the system and it increases with the earthquake intensity. Unlikely, this phenomenon could not be properly captured by numerical models, because it was not properly measured in the tests and has never been happened in the previous research.

**Figure 9:**
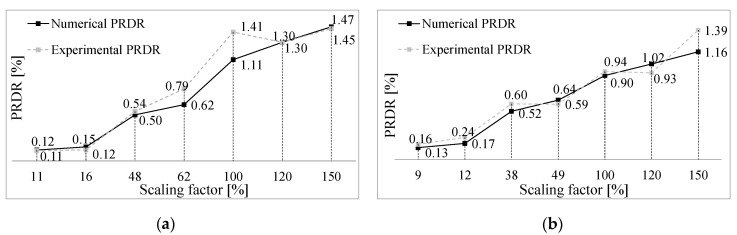
Comparison between experimental and numerical peak roof drift ratio (PRDR). (**a**) Type 1 mock-up; (**b**) Type 2 mock-up.

Moreover, a comparison between experimental and numerical results in terms of PRDR is shown in Figure 9. The experimental to numerical ratio is in the range 0.99 to 1.27 for Type 1 mock-up and 0.92 to 1.41 for Type 2 mock-up. It can be noticed that globally models are able to capture experimental behavior in term of roof drift ratio (RDR), but the PRDRs are not captured very accurately. The inaccuracy of the prediction could be attributed to the fact that models well predict the first mode, but with less accuracy the others.

## 6. Conclusions

The paper describes the numerical modelling developed to reproduce the dynamic and seismic behavior of two CFS strap-braced building, tested at University of Naples “Federico II”, as a part of a research project in cooperation with Lamieredil S.p.A. company. In particular, two three-story mock-ups in reduced scale (1:3), which differed only for the type of floors (composite steel-concrete and wood-based panels), were tested on shake-table. The two mock-ups were designed and opportunely scaled to be compatible with the available shake-table. Two types of signals were used for the dynamic identification and the seismic response evaluation: White noise signal and earthquake records, respectively.

Three-dimensional numerical models for both mock-ups were developed in OpenSees software to reproduce the experimental response. The models were calibrated on the base of available experimental data and validated through the comparison between experimental and numerical results obtained. In particular, the comparison between experimental and numerical dynamic properties was done in term of fundamental periods and modal shapes, whereas the seismic behavior was compared in term of peak inter-story drift and peak roof drift. Developed models captured with enough accuracy fundamental period of vibration (experimental-to-numerical ratio equal to 1.08 and 1.00 for Type 1 and Type 2 mock-ups, respectively) and modal shapes of mock-ups. Experimental and numerical peak inter-story drift ratios and roof drift ratios were compared, exhibiting experimental to numerical ratios in the range 0.80 to 2.08 and 0.80 to 1.27 for Type 1 mock-up and 0.65 to 2.06 and 0.91 to 1.41 for Type 2 mock-up. Models were able to capture peak inter-story drift ratios for low inputs, whereas they did not show good predictions for higher inputs. The reason behind the inaccurate response at high intensity earthquakes could be found in the adding source of deformability due to the rocking phenomenon observed experimentally. Unfortunately, since this phenomenon never happened in previous experimental campaigns, no ad-hoc instrumentation was installed for the monitoring and this makes very difficult the rocking modelling, which would be the focus of future studies.

## Figures and Tables

**Table 1:** Structural element properties (lengths in mm).

Component		Element	Properties
Wall	1st story	Stud	C50 × 20 × 10 × 0.7
		Track	U51.4 × 20 × 0.7
		Diagonal strap	80 × 0.7
		Gusset plates	130 × 130 × 0.7
		Track reinforcement	C50 × 20 × 10 × 0.7
		Blocking	C50 × 20 × 10 × 0.7 U51.4 × 20 × 0.7
		Flat strap	20 × 0.7
		Hold-down to chord stud fasteners	no. 4 M6 bolts Class 8.8
		Hold-down to steel beam fasteners	no. 1 M8 Class 10.9
	2nd story	Stud	C50 × 20 × 10 × 0.7
		Track	U51.4 × 20 × 0.7
		Diagonal strap	53 × 0.7
		Gusset plates	117 × 117 × 0.7
		Track reinforcement	C50 × 20 × 10 × 0.7
		Blocking	C50 × 20 × 10 × 0.7 U51.4 × 20 × 0.7
		Flat strap	20 × 0.7
		Hold-down to chord stud fasteners	no. 4 M6 bolts Class 8.8
		Wall to wall fasteners	no. 1 M8 Class 10.9
	3rd story	Stud	C50 × 20 × 10 × 0.5
		Track	U51 × 20 × 0.5
		Diagonal strap	55 × 0.5
		Gusset plates	100 × 100 × 0.5
		Track reinforcement	C50 × 20 × 10 × 0.5
		Blocking	C50 × 20 × 10 × 0.5 U51 × 20 × 0.5
		Flat strap	20 × 0.5
		Hold-down to chord stud fasteners	No. 4 M5 bolts Class 8.8
		Wall to wall fasteners	no. 1 M8 Class 10.9
Floor	composite	Joist	C85 × 20 × 10 × 0.7
		Corrugated sheet (thickness)	0.04
	wood-based	Joist	C85 × 20 × 10 × 0.7
		OSB panel	760 × 400 × 9

**Table 2:** Scaling factors for earthquake records for both mock-ups.

Mock-Up	Test Label	Time History Scaling Factor
Type 1	TH1, TH2, TH3, TH4, TH5, TH6, TH7	9%, 12%, 38%, 49%, 100%, 120%, 150%
Type 2	TH1, TH2, TH3, TH4, TH5, TH6, TH7	11%, 16%, 48%, 62%, 100%, 120%, 150%

**Table 3:** Comparison in term of fundamental periods of vibration.

	Type 1 Mock-Up			Type 2 Mock-Up		
	Exp	Num	Exp/Num	Exp	Num	Exp/Num
T1 [s]	0.43	0.40	1.08	0.50	0.50	1.00
T2 [s]	0.15	0.12	1.25	0.18	0.13	1.38
T3 [s]	0.10	0.07	1.43	0.11	0.08	1.38

**Table 4:** Comparison in term of fundamental periods of vibration.

Test Label	Peak Inter-Story Drift [%]											
	1st Level				2nd Level				3rd Level			
	Type 1		Type 2		Type 1		Type 2		Type 1		Type 2	
	EXP	NUM	EXP	NUM	EXP	NUM	EXP	NUM	EXP	NUM	EXP	NUM
TH1	0.10	0.07	0.12	0.07	0.13	0.14	0.17	0.13	0.18	0.16	0.31	0.13
TH2	0.13	0.09	0.22	0.09	0.15	0.21	0.28	0.15	0.16	0.23	0.28	0.17
TH3	0.40	0.33	0.35	0.34	0.61	0.71	0.73	0.58	0.75	0.73	0.89	0.55
TH4	0.47	0.41	0.44	0.37	0.94	0.89	0.73	0.68	1.03	0.92	0.98	0.68
TH5	0.83	0.76	0.52	0.56	1.59	1.60	1.17	0.94	2.15	1.62	1.65	0.83
TH6	0.80	0.84	0.47	0.66	1.58	1.86	1.34	1.03	2.23	1.89	1.38	1.07
TH7	0.92	1.00	0.62	0.71	1.88	2.15	1.81	1.17	3.49	2.01	2.21	1.23

## Data Availability

Data sharing is not applicable to this article.
